# Supplementary material for: Usability of pulse oximeters used by community health and primary care workers as screening tools for severe illness in children under five in low resource settings: A cross-sectional study in Cambodia, Ethiopia, South Sudan, and Uganda
Source: PLOS Glob Public Health. 2023 Jul 18;3(7):e0001800. doi: 10.1371/journal.pgph.0001800 (PMC10353789; doi:10.1371/journal.pgph.0001800)
Supplement: S1 Checklist — (DOC) [file pgph.0001800.s001.doc]

STROBE Statement—Checklist of items that should be included in reports of ***cross-sectional studies***

|  | Item No | Recommendation |
| --- | --- | --- |
| **Title and abstract** | 1 | (*a*) Indicate the study’s design with a commonly used term in the title or the abstract  *🡪 Reported on page 1 (line 4)* |
|  |  | (*b*) Provide in the abstract an informative and balanced summary of what was done and what was found  *🡪 Reported on page 2 (lines 24-45)* |
| Introduction | | |
| Background/rationale | 2 | Explain the scientific background and rationale for the investigation being reported  *🡪Reported from page 3-5 (lines47-100)* |
| Objectives | 3 | State specific objectives, including any prespecified hypotheses  *🡪Reported from page 4-5 (lines 97-100)* |
| Methods | | |
| Study design | 4 | Present key elements of study design early in the paper  *🡪Reported on page 5 (lines 102-110)* |
| Setting | 5 | Describe the setting, locations, and relevant dates, including periods of recruitment, exposure, follow-up, and data collection  *🡪Reported on pages 6 and 7 (lines 113-150) supported by Figure 1* |
| Participants | 6 | (*a*) Give the eligibility criteria, and the sources and methods of selection of participants  *🡪* *Reported on page 7 (lines 135-150) supported by Figure 1* |
| Variables | 7 | Clearly define all outcomes, exposures, predictors, potential confounders, and effect modifiers. Give diagnostic criteria, if applicable  *🡪Reported on page 8 (lines 163-178)* |
| Data sources/ measurement | 8* | For each variable of interest, give sources of data and details of methods of assessment (measurement). Describe comparability of assessment methods if there is more than one group  *🡪Reported on page 6 (lines 124-133), pages 8-9 (lines 163-200)*  *🡪Additional information in Figure 1* |
| Bias | 9 | Describe any efforts to address potential sources of bias  *🡪Reported on page 6 (lines 123-133), page 9 (lines 207-209), page 16 (lines 344-348)* |
| Study size | 10 | Explain how the study size was arrived at  *🡪Reported on pages 7 and 8 (lines 153-161)*  *🡪Supported by Figure 1* |
| Quantitative variables | 11 | Explain how quantitative variables were handled in the analyses. If applicable, describe which groupings were chosen and why  *🡪Reported on pages 8 and 9 (lines 163-200)* |
| Statistical methods | 12 | (*a*) Describe all statistical methods, including those used to control for confounding  *🡪 Reported on pages 8 and 9 (lines 180-200)* |
|  |  | (*b*) Describe any methods used to examine subgroups and interactions  *🡪 Reported on pages 8 and 9 (lines 180-200)* |
|  |  | (*c*) Explain how missing data were addressed  *🡪 Reported on pages 8 and 9 (lines 180-200) and page 16 (lines 344-348)* |
|  |  | (d) If applicable, describe analytical methods taking account of sampling strategy  *🡪 pages 8 and 9 (lines 180-200), page 16 (lines 351-354)* |
|  |  | (*e*) Describe any sensitivity analyses  *🡪 not applicable* |
| Results | | |
| Participants | 13* | (a) Report numbers of individuals at each stage of study—eg numbers potentially eligible, examined for eligibility, confirmed eligible, included in the study, completing follow-up, and analysed  *🡪 Reported in Figure 1* |
|  |  | (b) Give reasons for non-participation at each stage  *🡪 Reported on pages 6 and 7 (lines 124-150)* |
|  |  | (c) Consider use of a flow diagram  *🡪 Figure 1* |
| Descriptive data | 14* | (a) Give characteristics of study participants (eg demographic, clinical, social) and information on exposures and potential confounders  *🡪 Reported in Figure 1* |
|  |  | (b) Indicate number of participants with missing data for each variable of interest  *🡪Reported on page 10 (line 225)*  *🡪 Supporting material, S1 Table and S2 Table* |
| Outcome data | 15* | Report numbers of outcome events or summary measures  *🡪Reported on page 11 (tables 2 and 3), page 12 (tables 4 and 5)* |
| Main results | 16 | (*a*) Give unadjusted estimates and, if applicable, confounder-adjusted estimates and their precision (eg, 95% confidence interval). Make clear which confounders were adjusted for and why they were included  *🡪Reported on pages 10-12 (throughout tables 2-5)* |
|  |  | (*b*) Report category boundaries when continuous variables were categorized  *🡪not applicable* |
|  |  | (*c*) If relevant, consider translating estimates of relative risk into absolute risk for a meaningful time period  *🡪not applicable* |
| Other analyses | 17 | Report other analyses done—eg analyses of subgroups and interactions, and sensitivity analyses  *🡪Reported on pages 10 and 11 (lines 233-235, table 3)* |
| Discussion | | |
| Key results | 18 | Summarise key results with reference to study objectives  *🡪Reported on page 12 and 13 (lines 259-264)* |
| Limitations | 19 | Discuss limitations of the study, taking into account sources of potential bias or imprecision. Discuss both direction and magnitude of any potential bias  *🡪Reported on pages 15 and 16 (lines 326-354)* |
| Interpretation | 20 | Give a cautious overall interpretation of results considering objectives, limitations, multiplicity of analyses, results from similar studies, and other relevant evidence  *🡪Reported on pages 13 to 15 (lines 265-324) and page 16 (lines 356-360)* |
| Generalisability | 21 | Discuss the generalisability (external validity) of the study results  *🡪Reported on page 15 (line 324)* |
| Other information | | |
| Funding | 22 | Give the source of funding and the role of the funders for the present study and, if applicable, for the original study on which the present article is based  *🡪Funding information provided in journal submission document as requested by the journal* |

*Give information separately for exposed and unexposed groups.

**Note:** An Explanation and Elaboration article discusses each checklist item and gives methodological background and published examples of transparent reporting. The STROBE checklist is best used in conjunction with this article (freely available on the Web sites of PLoS Medicine at http://www.plosmedicine.org/, Annals of Internal Medicine at http://www.annals.org/, and Epidemiology at http://www.epidem.com/). Information on the STROBE Initiative is available at www.strobe-statement.org.
