# Supplementary material for: Usability of pulse oximeters used by community health and primary care workers as screening tools for severe illness in children under five in low resource settings: A cross-sectional study in Cambodia, Ethiopia, South Sudan, and Uganda
Source: PLOS Glob Public Health. 2023 Jul 18;3(7):e0001800. doi: 10.1371/journal.pgph.0001800 (PMC10353789; doi:10.1371/journal.pgph.0001800)
Supplement: Attachment — Submitted filename: Response to Reviewers.pdf [file pgph.0001800.s007.pdf]

Sakib Burza
Academic Editor
PLOS Global Public Health

May 26th , 2023

Dear Sakib,
Dear Reviewers,

Thank you all for rereading our manuscript and your continued commitment in further improving our manuscript. You find attached here our revised manuscript including all changes we made following your remaining suggestions and comments. Please note that line numbers refer to the tracked changes version of the newly revised manuscript.

Reviewer 1

We are happy to have been able to address most of your comments in the previous revision and your continued support and commitment for our manuscript. You can find our responses to your remaining comments below and the adjustments in the revised manuscript. Thank you again for your thoughtful input to our work and helping shape the messaging and improve the quality of the publication, it is much appreciated. We lastly hope to have been able to address all your concerns.

I could not see the STROBE checklist – perhaps this was in another part of the submission or omitted by mistake?

Apologies for this, we failed to include the STROBE checklist on the submission platform. However, we did forward the checklist to the journal via email, unfortunately it seems there have been problems in passing it on. It should be included in the re-submission now though. However please note that line notations mentioned in the checklist refer to line numbers in the first revision version of the manuscript.

I am in agreement with Reviewer 3 that the overall tone of the manuscript should perhaps be moderated with regards the positivity of the findings, in particular with regards the usability of inaccurate (and not recommended) finger-tip devices. In Line 83, please clarify that the satisfactory performance of the pulse oximeters relates to handheld devices and that finger-tip device performance was poor in the cited study (reference 15), which concluded that they should not be used in children under the age of five. Perhaps it would also be worth justifying why the authors think it is helpful to report usability of inaccurate and non-recommended devices? I can see (and agree) that probably this is because the data have been collected and results should be presented (partly to avoid repetition by other groups) but it might be helpful to explain that their assessment of usability of the finger-tip devices does not imply a change in the position of the authors and they (still) do not recommended the use of finger-tip devices in children under the age of five.

Thank you for your suggestion. In lines 80-83 of the newly revised manuscript we have clarified that performance, as well as operational feasibility testing results pertain to handheld devices. We have made further adjustments in lines 106-107 to avoid confusions about performance results. As your rightly mentioned data on usability has been collected before performance results were analysed and published and we believe that it is nonetheless useful to report findings on all collected data, even though performance results of fingertip pulse oximeters were poor. Additionally, we believe that it is important to report the usability results of fingertip devices, since these results can help to inform future pulse oximeter device designs together with the performance results and help to develop devices

specifically suited for lower levels of low-resource health care settings. We have added this justification in lines 107-109. In order to clarify that the results of the usability study do not imply a change in our position but rather refine our recommendations of handheld devices towards devices with more usable universal probes we have made some adjustments in the discussion section lines 298 -305.

Related to the above, Lines 110-111 suggest that the performance of the finger-tip devices was deemed satisfactory, whereas I think actually only the performance of the handheld devices was deemed satisfactory (reference 15). Perhaps the authors are referring to the fact that all five devices had passed laboratory testing? In line with the above point, this should be clarified, to avoid confusion amongst readers that the authors are now recommending the finger-tip devices on the basis of satisfactory performance?

We believe this point has been addressed by the changes explained above. In the revised manuscript you find a corrected statement regarding performance results of device types and further explanations on the inclusion of all device types for usability analyses in lines 106-109.

I note the comments from Reviewer 4 with regards missingness along with my earlier comments. I understand that multiple imputation may not have been appropriate due to missingness being concentrated in particular study locations but I am still not clear why listwise rather than pairwise deletion has been used? For participants/observations in which data on some (but not all) items in the checklist were available, why not include these data? The overall checklist completion rate could still be reported as it is currently. Is there a reason why data recorded for individual checklist items within incomplete checklists might be unreliable? If not, I cannot see a reason why pairwise deletion shouldn't be used? I would at least want to see this approach as a sensitivity analysis to show it doesn't change the conclusions, particularly as the authors spend much of the manuscript discussing and interpreting the overall findings of the study in the context of individual checklist item completion rates. Hence, it is very important to be sure that the estimates for individual checklist item completion rates are not biased by the way missing data have been handled.

We understand your persisting concern about the pattern of missing data since it can potentially affect conclusions. With regards to an overall checklist completion rate we believe that pairwise deletion is not appropriate since the checklist completion rate is a binary outcome defined as full checklist adherence vs. not full checklist adherence (lines 190-191), thus for observations with missing data in one or more checklist steps, full adherence cannot be determined. We present the rationale for our choice in lines 280-282. We preferred listwise to pairwise deletion also for task completion rates of single checklist items for two main reasons. First, there are observations in the dataset that have a lot of missing data in the checklist steps, e.g. they might only have one checklist step recorded. This constitutes a concern whether the coding is reliable if so much data is missing in one observation. The second reason is to guard consistency within statistical analysis. However, as you rightfully pointed out it is important to ensure that the estimates for individual checklist item completion rates are not biased by the exclusion of observations with missing data. Therefor we have performed the sensitivity analyses you have asked for and included results on task completion rates per checklist item using pairwise deletion in S4 Table, and refer to this in lines 366-369 of the manuscript. As you can see from S4 Table, there is no major change in task completion rates per checklist item and it does not affect the main conclusions including that Step4 constitutes the checklist step with the lowest task completion rate, suggesting that choosing the correct probe for the child's age constitutes a difficulty in using handheld pulse oximeters with multiple probes.

I do not agree that it is not possible to explore whether the different completion rates in infants is driven by the difficulties in selecting the correct probe (Line 313) – if all (or a disproportionate amount) of incomplete checklists amongst infants is due to this item compared to older children (i.e.

the association with age is confounded by device type [as probe selection only relates to the handheld devices]) that would support the author's conclusions that a single-probe device would have a great advantage – and be reassuring (although admittedly perhaps not definitively so depending on power) that there is not some other factor at play with regards to why infants had lower completion rates. This is important because it lends credibility to the author's overall recommendations that research and implementation of single-probe handheld devices should be prioritized. At the moment the tone of the discussion and analyses is that probe selection and age category are two separate issues: Line 273 – "also revealed"; Line 294 – "additionally". However, I think the data are there to unpack this further to some degree.

Thank you for further explaining your concerns regarding the findings on differences between device groups and age groups. We did not perform correlation tests and our dataset unfortunately does not contain sufficient information to build a meaningful regression model that shows a satisfactory fit. Thus, we stated in the manuscript that the tests performed in this analysis do not provide sufficient information for conclusions to be drawn on a correlation between device type and child age effect. We acknowledge the value of further exploring this relationship and have thus added information on task completion rates of Step 4 by age group in lines 245-247, results section. Additionally, we provide task completions rates of all checklist items by age group in the supplementary material (S3 Table). You can find further discussion of those results in lines 323-330.

Table 2 – missing frequency in the handheld devices row.

Thanks for catching this, adjusted.

Line 309 – doesn't make sense – do you mean "should be accelerated"?

Thanks for pointing out, we see that this phrase is not clear, see lines 305-306 for a correction.

Line 303 – as previously mentioned, I tend to agree with Reviewer 3 that generally there could be stronger messaging about the good usability findings of the finger tip devices being contextualized with their poor performance. The cited trial from your group actually recommends they are not used in children under five. Think this could be stated rather than simply saying the handheld devices performed better.

As mentioned in our response to your second comment we have adjusted lines 301-308 to better reflect the contrast between usability and performance findings and provide a discussion of this contrast's implications on our recommendations.

Reviewer 4

We are happy to have been able to address most of your concerns and comments in the last revision round. We have included your two remaining suggestions in the newly revised manuscript and you can find answers to the specific comments below. Thank you again for your thoughtful input to our work and helping shape the messaging and improve the quality of the publication, it is much appreciated. We lastly hope to have been able to address all your concerns.

You wrote in your response: "The Satisfaction Score we built is a summated score that was subsequently standardized to a value between 0 and 100. Thus, if we would assume someone had rated all 19 Likert items with a score of 1 this would pertain to a Satisfaction Score of 20 ((19*1)*100/(19*5)). You can find this explanation in a shortened version in lines 185-190."

My new response: This means the score is actually between 20 and 100 as it's not possible to score

lower than 20 is it? Please correct methods line 187 to read “value between 20 and 100”. You could also add your explanation above to the paper to make it clear that the minimum score possible of all 1s would score 20 and the maximum possible score of all 5s would score 100.

Thank you for catching this we have corrected the explanations in lines 190-193 of the newly revised manuscript (tracked changes version) and added the explanation and formula from above.

You wrote in your response: "As you rightfully pointed out the missing data was concentrated in Uganda which also supports our conclusion that there were difficulties in the data collection process. The substantial amount of missing data limits the informative value of the results for Uganda since the sample is small."

My new response: Thanks – would be good to mention Uganda specifically on this point in the discussion – i.e. the sentence ending on line 347 could continue to say something like: “problems during the data collection process, especially in Uganda where most of the missing data was concentrated (Table S1)”

Thanks for the suggestion. We have added this information in lines 355-356 of the newly revised manuscript (tracked changes version) and refer to Table S1.

We hope to have been able to address all remaining concerns. We are looking forward to hearing back from you.

Best Regards

Dr. Theresa Pfurtscheller
