# Supplementary material for: Usability of pulse oximeters used by community health and primary care workers as screening tools for severe illness in children under five in low resource settings: A cross-sectional study in Cambodia, Ethiopia, South Sudan, and Uganda
Source: PLOS Glob Public Health. 2023 Jul 18;3(7):e0001800. doi: 10.1371/journal.pgph.0001800 (PMC10353789; doi:10.1371/journal.pgph.0001800)
Supplement: Attachment — Submitted filename: Response to Reviewers.docx [file pgph.0001800.s006.docx]

Sakib Burza
Academic Editor
PLOS Global Public Health

March 12^th^, 2023

Dear Sakib,

Thank you for your response on our submission and for reading our manuscript and providing valuable feedback. You find attached here our revised manuscript including all changes we made following your suggestions and comments. The data used for this study has been made openly available on figshare (Pfurtscheller, Theresa et al. (2022) figshare. Dataset. <https://doi.org/10.6084/m9.figshare.21764123.v1>). Below please find additional responses to the reviewer’s comments.

Reviewer 1

Thank you so much for your appreciation of our work and the feedback. Within this revised submission we include the STROBE checklist for cross-sectional studies. We have followed this checklist but had not submitted it with our manuscript. We have included your suggestions for the introduction section. With regards to the choice of listwise rather than pairwise deletion, we have decided on a full case analysis based on the pattern of missing data where most of the missing data belongs to some individuals. We think this is related to problems during data collection. We have clarified this in the discussion section of the manuscript as well (lines 345-348). We included numerators (Frequency) and denominators (N) for task completion rates in Table 2 and 3. In Table 3 we have also clarified which steps of the checklist pertain to which devices. We apologize for the confusion regarding the statement of overlapping confidence intervals between handheld devices which is indeed not supported by Table 3. While we have conducted sensitivity analyses per device we have decided to report results on differences between groups only for the device type groups based on our sample size, thus we have removed the statement that refers to differences between single devices. This might also help to explain our statement of slight variations in satisfaction between devices which you highlighted in the discussion section. This statement also referred to a difference found between single devices, which is not the focus and scope of this manuscript, we have thus removed it. To help readers better contextualize the performance findings with the usability results of this study we have summarised important aspects of the performance study in lines 288-294. The question about correlations between differential effectiveness rates in younger children and the device type is very interesting. However, the tests performed in our analysis do not allow for conclusions to be drawn about correlations between the two. Previous research has indicated that pulse oximeters are more difficult to use in younger children and our study highlights the need for further research on the root cause for this and whether it is related to device type. We have clarified this in the discussion section (line 295-307). We have removed varying altitude levels of study sites from the discussion section, as you rightfully raised the question on the role of altitude levels for usability. Altitude levels affect hypoxemia definitions and Sp02 cut offs, however they do not have an impact on whether device’s effectiveness, efficiency or health worker’s satisfaction. Decimal points have been standardized throughout the document. Unfortunately, we do not have data on the refusal rate of health care workers or care takers available to us. We hope we were able to resolve your concerns and clarify some outstanding issues. Thank you for your valuable input!

Reviewer 2

Thank you so much for your appreciation of our work and the feedback. We have revised our manuscript mainly in the methodology, results and discussion section to clarify choices of methodology and statistics and work out the messaging of our conclusions more. We hope by this we were able to resolve any outstanding concerns. Thank you for your valuable input!

Reviewer 3

Thank you so much for your thoughts and feedback on our work! We acknowledge your concerns related to the messaging of our findings and agree with you on the importance of highlighting a need for development and research on usability in the age group <12 months. We have revised our discussion section strengthening the argument on low usability of the most accurate devices and in children under one as a highly important age group. Apart from this call to action we do feel it is important to highlight as well that task completion rates were very high in all steps except for Step 4 that related to probe choice. Together with previous research showing a substantial impact of pulse oximetry on child health outcomes and the results from a performance study that showed that community health workers can measure Sp02 accurately with pulse oximeters this finding supports scaled use of pulse oximeters at lower health care levels in low resource settings. We hope to have clarified that pulse oximeters can be usable, accurate and can greatly help to increase health outcomes of children under five in these settings, given that further development and research are put into specific devices and age groups. Unfortunately, we do not have any data available on <3 measurement tries. We acknowledge your concerns on unobserved situations but are unable to deduct any information on this from our study data. Your concerns regarding social desirability bias and the Hawthorne effect are of course understandable. We have tried to minimize these by choosing random days of observation over a period of three months. As the locations of the study in community health services are fairly remote and we only observed health workers on the days of field visits there is no data available on the percentage of eligible patients that had measurements done overall. However, as this study was part of a multi-centre study containing other diagnostic devices we can say that in a sample of over 1000 children all children with difficulty breathing and/or cough had pulse oximetry measurements recorded and even an additional 18 children that did not fulfil this eligibility criterion had measurements recorded. Our results show high satisfaction of health workers with the devices which also supports the conclusion that they are eager to use these additional devices and feel that the devices support their daily work rather than pose an additional burden on them. We hope that we were able to address your concerns and clarify some outstanding issues, thank you for your valuable input that has helped strengthened the quality of our work.

Reviewer 4

Thank you so much for your thorough review of our manuscript and the feedback. We have clarified in the abstract that fingertip devices are single-probe devices and the handheld devices in this study are multi-probe devices which should help readers to better understand the recommendation of prioritizing single-probe handheld devices. We acknowledge that the distinction of main outcomes has caused confusion in the methods section. Initially this was referring to the more extensive variables for effectiveness and satisfaction analyses, however we have rephrased this and taken out the distinction to avoid confusion. With regards to missing data, you can now find the proportion of missing data for effectiveness analyses (efficiency and satisfaction did not show missing data) in line 225 and a breakdown of missing values across checklist steps in the supplementary material S2 Table. We decided to use listwise deletion and to not impute data due to the missing data pattern where most of the missing data belonged to some individuals. The individuals that have missing data tended to have a lot of missing data which we think is related to problems during the data collection process and using multiple imputation for individuals with scarce data is not recommended since multiple imputation is based on having access to data on other variables for that individual, if this is not the case the imputed data would end up to be some kind of average value for the whole sample. We have tried to further clarify and explain this with additional reference in the discussion section (line 344-348). As you rightfully pointed out the missing data was concentrated in Uganda which also supports our conclusion that there were difficulties in the data collection process. The substantial amount of missing data limits the informative value of the results for Uganda since the sample is small. However, in this study different geographic locations and slightly different income levels have helped the transferability of the results across low resource settings and while the results for Uganda alone are of limited value they do support the analysis and conclusions in whole. The Satisfaction Score we built is a summated score that was subsequently standardized to a value between 0 and 100. Thus, if we would assume someone had rated all 19 Likert items with a score of 1 this would pertain to a Satisfaction Score of 20 ( (19*1)*100/(19*5) ). You can find this explanation in a shortened version in lines 185-190. Regarding your question on regression analyses to look for associations we want to point out that our main aim with this manuscript has not been to assess potential associations. However, in an early version we also included some regression analysis that we left out later to keep the focus of the paper and not overload it with tables. We also concluded that the regression analysis did not add important conclusions. Thank you for the reference and pointing out that no overlapping confidence do of course not always exclude statistical significance. We apologize for the confusion on this statement since these analyses are not shown in our tables. In an earlier version we had included analyses for differences between single devices. Given our sample size and the relevance for conclusions we have however shifted our focus on differences between device types and have thus now excluded this sentence. We have included efficiency results in Table 4 now. Thank you for raising the relation between severe illness, low perfusion and difficulties in the measurement process as this is very relevant and interesting. Unfortunately, in our data we were unable to relate Sp02 levels/hypoxemia to longer or more complicated measurement processes since health workers were given a fixed amount of tries for their measurements but it was not recorded how many times they needed to try. We agree that differing prevalence of hypoxemia between study sites could impact differences between devices. However, all devices were used in all countries of the study and devices were assigned randomly to health workers within countries. We hope that we have been able to clarify outstanding issues and address your concerns especially regarding information on methodological and statistical choices. Thank you so much for your valuable input that we think has helped to improve our manuscript for publication.

We hope to have been able to address all outstanding concerns. We are looking forward to hearing back from you.

Best Regards

Dr. Theresa Pfurtscheller
